# Size-Optimized Microspace Culture Facilitates Differentiation of Mouse Induced Pluripotent Stem Cells into Osteoid-Rich Bone Constructs

**DOI:** 10.1155/2020/7082679

**Published:** 2020-05-14

**Authors:** Phoonsuk Limraksasin, Hiroko Okawa, Maolin Zhang, Takeru Kondo, Thanaphum Osathanon, Prasit Pavasant, Hiroshi Egusa

**Affiliations:** ^1^Division of Molecular and Regenerative Prosthodontics, Tohoku University Graduate School of Dentistry, Sendai, Miyagi 980-8575, Japan; ^2^Weintraub Center for Reconstructive Biotechnology, UCLA School of Dentistry, Los Angeles, CA 90095-1668, USA; ^3^Center of Excellence for Regenerative Dentistry and Department of Anatomy, Faculty of Dentistry, Chulalongkorn University, Bangkok 10330, Thailand; ^4^Center for Advanced Stem Cell and Regenerative Research, Tohoku University Graduate School of Dentistry, Sendai, Miyagi 980-8575, Japan

## Abstract

Microspace culture is promising for self-organization of induced pluripotent stem cells (iPSCs). However, the optimal size of microspaces for osteogenic differentiation is unclear. We hypothesized that a specific microspace size could facilitate self-organizing iPSC differentiation to form bone-like tissue *in vitro*. The objectives of this study were to investigate such effects of microspace size and to evaluate bone regeneration upon transplantation of the resulting osteogenic constructs. Dissociated mouse gingival fibroblast-derived iPSCs were plated in ultra-low-attachment microspace culture wells containing hundreds of U-bottom-shaped microwell spots per well to form cell aggregates in growth medium. The microwells had different aperture diameters/depths (400/560 *μ*m (Elp400), 500/700 *μ*m (Elp500), and 900/700 *μ*m (Elp900)) (Kuraray; Elplasia). After 5 days of aggregation, cells were maintained in osteogenic induction medium for 35 days. Only cells in the Elp500 condition tightly aggregated and maintained high viability during osteogenic induction. After 10 days of induction, Elp500 cell constructs showed significantly higher gene expression of *Runx2*, *Osterix*, *Collagen 1a1*, *Osteocalcin*, *Bone sialoprotein*, and *Osteopontin* compared to constructs in Elp400 and Elp900. In methylene blue-counterstained von Kossa staining and Movat's pentachrome staining, only Elp500 constructs showed robust osteoid formation on day 35, with high expression of type I collagen (a major osteoid component) and osteocalcin proteins. Cell constructs were transplanted into rat calvarial bone defects, and micro-CT analysis after 3 weeks showed better bone repair with significantly higher bone mineral density in the Elp500 group compared to the Elp900 group. These results suggest that microspace size affects self-organized osteogenic differentiation of iPSCs. Elp500 microspace culture specifically induces mouse iPSCs into osteoid-rich bone-like tissue possessing high bone regeneration capacity.

## 1. Introduction

Extensive loss of bone following trauma, tumor resection, or long-term tooth loss in dentistry exceeds the natural healing capacity, and bone regeneration in large defects is a significant challenge in the clinic [1]. Although bone regeneration using autologous bone grafts is the gold standard, harvesting of the graft requires an invasive surgical procedure and is associated with donor site morbidity [2]. Stem cell-based bone tissue engineering is an alternative approach to eliminate the drawbacks of current clinically used treatments, particularly donor site morbidity and limited availability [3]. In addition, this approach has osteoinductive properties that are crucial for efficient bone repair in clinical applications [4]. Stem cell-based therapy approaches in bone regeneration have been under development for years and generally involve growing mesenchymal stem cells (MSCs) on biomaterial scaffolds enriched with growth factors. However, such approaches have not been able to achieve complete bone healing in large defects due to fibrous tissue encapsulation, degradation of engineered tissue, immune responses to the scaffold material [3, 5], and migration and death of the transplanted MSCs [6]. Therefore, there is an unmet need for effective protocols for efficient bone tissue engineering to achieve sufficient regeneration.

Induced pluripotent stem cells (iPSCs) derived from reprogramming of somatic cells [7] have self-organizing potential that contributes to three-dimensional (3D) tissue or organ construction without requiring the use of a scaffold; thus, iPSCs could be a promising source to generate tissue-engineered bone. Previous studies demonstrated *in vitro* osteogenic differentiation of iPSCs in 2D adherent culture [8–10]. We previously fabricated iPSC-derived 3D-osteogenic constructs with high expression of osteocalcin, a crucial extracellular matrix (ECM) molecule for bone formation [11]. However, the inner region of the fabricated constructs showed central necrosis and thus might not be suitable for clinical application. In addition, the osteogenic constructs showed *in vivo* teratoma formation, suggesting incomplete osteogenic differentiation.

The microenvironment of stem cells, including aspects of the stem cell niche such as growth factors, cell-cell contact, and cell-matrix interactions, has been reported to govern stem cell fate and behavior. The translation of stem cell-based therapies to treat degenerated tissue relies on stem cell lineage commitment in the region of interest, in which the microenvironment precisely controls the commitment and success [12]. This microenvironment concept has been applied to promote stem cell commitment toward osteogenic lineages in coculture [13, 14] and 3D cell-scaffold culture systems [15, 16]. The formation of iPSC aggregates, i.e., the so-called embryoid bodies (EBs), prior to differentiation provides *in vitro* microenvironments for stem cells and influences multiple pathways that may control the differentiation trajectory [17, 18]. Several methods have been developed to form and culture iPSC aggregates. Among them, microspace culture, in which iPSCs in different microspaces accumulate and then form aggregates, could be a candidate for tissue engineering [19] to provide a large number of homogenous iPSC aggregates in a less time-consuming manner [20]. Recently, Takebe et al. achieved massive and reproducible production of 3D liver bud organoids from iPSCs using microspace culture plates [21]. Therefore, microspace culture may represent a promising microplatform to facilitate self-organizing differentiation of iPSCs by providing an appropriate microenvironment for bone tissue engineering. In addition, the size of the microspace has been reported to affect the differentiation potential of pluripotent stem cells [22, 23]. However, effects of microspace size on osteogenic differentiation of 3D-iPSC constructs have not yet been investigated.

In this study, we used microspace well plates (Elplasia; Kuraray) to fabricate and culture iPSC aggregates during osteogenic differentiation. We hypothesized that a specific microspace size could facilitate self-organizing differentiation of iPSCs to form bone-like tissue *in vitro*. The aims of this study were to investigate such effects of microspace size and to evaluate bone regeneration upon transplantation of the resulting osteogenic iPSC constructs.

## 2. Materials and Methods

### 2.1. iPSCs

Mouse iPSCs that had been previously generated using retroviral introduction of Oct3/4, Sox2, and Klf4 [7] were used in this study. iPSCs were maintained on mitomycin-C-treated SNLP76.7-4 feeder cells in growth medium (ES medium), which consisted of Dulbecco's modified Eagle's medium (DMEM with 4.5 g/l glucose and without sodium pyruvate; Nacalai Tesque, Kyoto, Japan), 15% FBS (Gibco/Life Technologies, Grand Island, NY, USA), 2 mM L-glutamine (Wako Pure Chemical, Osaka, Japan), 1 × 10^−4^ M nonessential amino acids (Life Technologies), 1 × 10^−4^ M 2-mercaptoethanol (Gibco/Life Technologies, Grand Island, NY, USA), 50 U of penicillin, and 50 *μ*g/ml streptomycin (Wako Pure Chemical).

### 2.2. Fabrication and Osteogenic Induction of 3D-iPSC Constructs

iPSCs were expanded on SNLP76.7-4 feeder cells in 6-well culture plates. After iPSCs reached confluence, trypsinization was performed. In brief, 500 *μ*l of 0.25% trypsin and 1 mM EDTA (Wako Pure Chemical) were applied to each well of the 6-well culture plates and maintained until detachment of SNLP76.7-4 feeder cells was observed. Next, the supernatant was aspirated to remove the SNLP76.7-4 feeder cells, and then, 500 *μ*l of trypsin solution was added to collect the iPSCs. The iPSCs were dissociated to single cells by gently pipetting the iPSC-trypsin suspension several times for 1 minute prior to preparing aliquoted single-cell suspensions in ES medium with concentrations of 1.95 × 10^6^ cells/ml and 3.9 × 10^6^ cells/ml. The viable iPSCs were counted using an EVE automatic cell counter (NanoEnTek, Guro-gu, Seoul, Korea) together with Trypan blue staining (NanoEnTek, Guro-gu).

3D-iPSC constructs were fabricated using ultra-low-attachment 6-well and 24-well microspace cell culture plates, which contain hundreds of U-bottom-shaped microwell spots per well [19, 21]. Three different aperture diameter widths and depths for the microwells were used, i.e., 400 *μ*m in width and 560 *μ*m in depth (Elp400; Cat. #RB 400 560 NA 6, Kuraray, Tokyo, Japan), 500 *μ*m in width and 700 *μ*m in depth (Elp500; Cat. #RB 500 700 NA 6 and Cat. #RB 500 700 NA 24, Kuraray), and 900 *μ*m in width and 700 *μ*m in depth (Elp900; Cat. #RB 900 700 NA 6 and Cat. #RB 900 700 NA 24, Kuraray). To eliminate bubbles at the bottom of each microwell, 1 ml and 2 ml of ES medium were added to each well of 24-well and 6-well plates, respectively; then, the plates were subsequently subjected to plate spin down (PlateSpinII, Kubota, Tokyo, Japan). Next, 1 ml of cell suspension (1.95 × 10^6^ cells/ml) was applied to each well in the 3D culture 24-well plates to form iPSC aggregates. For 3D culture 6-well plates, 2 ml of cell suspension (3.9 × 10^6^ cells/ml) was applied to each well (Supplementary Figure 1).

**Figure 1:**
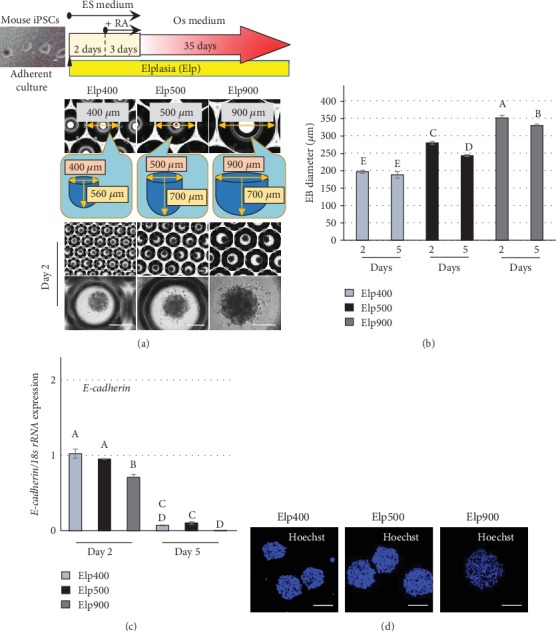
Microspace size influences the condensation of iPSC aggregates. (a) Fabrication and osteogenic induction of 3D-iPSC constructs. Representative images of iPSC aggregates were obtained under phrase-contrast microscopy after 2 days of aggregation in Elp400, Elp500, and Elp900. Scale bars: 200 *μ*m. (b) Size measurement of iPSC aggregates using ImageJ software (National Institutes of Health, Bethesda, MD, USA) to analyze Ferret's diameter at culture days 2 and 5. Different letters indicate significant differences between each group (*P* < 0.05, ANOVA with Tukey's multiple comparison test). The data represent the mean ± SD (*n* = 3). (c) Cell-cell adhesion marker gene expression with different microspace sizes. *E-cadherin* expression was determined by quantitative real-time RT-PCR at days 2 and 5. Different letters indicate significant differences between groups (*P* < 0.05, ANOVA with Tukey's multiple comparison test). The data represent the mean ± SD (*n* = 3). (d) Representative images of nuclear staining of iPSC aggregates using Hoechst. Scale bars: 200 *μ*m.

After 2 days of floating culture, 75% of the medium was aspirated from each well of 24-well and 6-well plates, followed by the addition of 0.5 ml and 1 ml of new ES medium that contained 1 *μ*M all *trans* retinoic acid (RA; Wako Pure Chemical), respectively. Then, half of the medium was replaced with new ES medium containing 1 *μ*M RA. This RA treatment condition was maintained for another 3 days, and half medium change was performed after 2 days. For osteogenic induction, the culture medium was replaced via half medium change performed twice with osteogenic induction medium (OS medium), consisting of *α*-MEM (Nacalai Tesque) that was supplemented with 15% FBS (Gibco/Life Technologies), 0.1 *μ*M dexamethasone (Sigma-Aldrich, St. Louis, MO, USA), 10 mM *β*-glycerophosphate (Sigma-Aldrich), 50 *μ*g/ml ascorbate-2-phosphate (Sigma-Aldrich), and 1% antibiotic-antimycotic (100 U/ml penicillin, 100 *μ*g/ml streptomycin, and 250 ng/ml amphotericin b; Gibco/Life Technologies) [8]. Half of the OS medium was changed every 2 days, and culture was maintained for 35 days. Day 5 of 3D culture refers to Day 0 of osteogenic induction commencement (Figure 1(a)). The constructs obtained from this induction are referred to as osteogenically induced iPSC (OI-iPSC) constructs.

### 2.3. Reverse-Transcription Polymerase Chain Reaction (RT-PCR)

The cultured 3D constructs were immediately frozen in liquid nitrogen before TRIzol extraction (Ambion/Life Technologies, Carlsbad, USA). Total RNA was isolated and purified using a spin column (RNeasy Mini Kit; Qiagen, GmBH, Germany) followed by DNase treatment and removal (DNA-free Kit; Invitrogen/Thermo Fisher Scientific, Vilnius, Lithuania). Complementary DNA was synthesized from 1 *μ*g of total RNA as previously described [11]. PCR was performed on a StepOnePlus Real-Time PCR system (Applied Biosystems, Thermo Fisher Scientific, Waltham, MA, USA) using the Thunderbird SYBR qPCR Mix (Toyobo, Osaka, Japan). The comparative cycle time (*△△*CT) method was used to quantitatively analyze the level of gene expression. The target gene expression levels were normalized to those of *18*s *rRNA*. The primer sequences used are shown in Supplementary Table 1.

### 2.4. Live/Dead Cell Viability Assay

The Live/Dead Viability/Cytotoxicity Kit (Molecular Probes/Thermo Fisher Scientific, Eugene, OR, USA) was used to assess the cell viability of iPSCs. After the iPSC constructs were maintained under osteogenic induction medium for 7 and 14 days, cells were washed with phosphate-buffered saline (PBS; Wako Pure Chemical) and incubated with the Live/Dead Viability/Cytotoxicity Kit for 30 minutes at room temperature. Subsequently, live cells (green fluorescence) and dead cells (red fluorescence) were detected by confocal microscopy (LSM780, Zeiss) using excitation/emission wavelengths of 494/517 nm and 528/617 nm, respectively. Incubation of iPSC constructs in 70% methanol for 30 minutes was used as a negative control.

### 2.5. Histochemical Analyses

OI-iPSC constructs were fixed with 10% neutral-buffered formalin solution (Wako Pure Chemical) for 1 day. Next, the specimens were embedded in paraffin, followed by the preparation of sequential sections with 4 *μ*m thickness using a microtome. Subsequently, the paraffin-embedded sections were evaluated using histological and immunofluorescent staining. The histological staining performed in this study was standard hematoxylin and eosin (HE) staining, methylene blue-counterstained von Kossa staining, and Movat's pentachrome staining. The paraffin-embedded sections were deparaffinized with xylene (Wako Pure Chemical) and hydrated through graded alcohol to distilled water prior to HE staining and methylene blue-counterstained von Kossa staining. For methylene blue-counterstained von Kossa staining, the specimens were incubated with 5% silver nitrate (AgNO_3_; Wako Pure Chemical) under ultraviolet (UV) light for 10 minutes, then rinsed in two changes of distilled water. Next, the slides were incubated in 5% sodium thiosulfate (Sigma-Aldrich) for 5 minutes to eliminate unreacted silver and washed with running water for 5 minutes. Subsequently, nuclear counterstaining was performed using 1% methylene blue (Wako Pure Chemical) in 10 mM borate buffer. For Movat's pentachrome staining, the staining was performed as previously reported [24–26].

### 2.6. Immunofluorescent Staining

Antigen retrieval was performed via incubation of specimens in a solution of 0.5 M acetic acid (Sigma-Aldrich) with 0.1% pepsin (Nacalai Tesque) at 37°C for 1 hour in a humid chamber prior to immunofluorescent staining. After washing, nonspecific binding was blocked for 60 minutes using 2% BSA (Wako Pure Chemical), 0.1% Tween 20 (Sigma-Aldrich), and 0.01% Triton-X (Wako Pure Chemical) prior to incubation in the primary antibody at 4°C overnight. After washing, the specimens were incubated with the secondary antibody for 1 hour at room temperature. Subsequently, fluorescence was detected by confocal microscopy (LSM780, Zeiss). The primary antibodies used in this study were anti-type I collagen monoclonal antibody (NB600-450: 1/50, Novus Biologicals, Littleton, CO, USA), anti-osteocalcin polyclonal antibody (FL-95: 1/50, Santa Cruz Biotechnology), and control IgG (normal mouse IgG (sc-2025): 1/50 or rabbit IgG (sc-2027): 1/50, Santa Cruz Biotechnology). Secondary antibodies were Alexa Fluor 488-conjugated goat anti-mouse IgG (1/500, Molecular Probes, Thermo Fisher Scientific) or Alexa Fluor 555-conjugated goat anti-rabbit IgG (1/500, Thermo Fisher Scientific). Nuclear staining was performed using Hoechst 33258 (1/500, Thermo Fisher Scientific).

### 2.7. Animal Experiments

All animal experiments in this study strictly followed a protocol approved by the Animal Research Subjects Committee of Tohoku University (approval number: 2018DnA-022). The animal experiments were modified from a previous method [27]. In total, 8 male 10-week-old Sprague-Dawley (SD) rats (Nippon SLC, Shizuoka, Japan) were used for the study. The animals were subjected to anesthesia, and critical-size calvarial-bone defects (5 mm in diameter) were created in the right and left parietal regions. Then, the left and right defects were filled with living OI-iPSC constructs prepared with Elp500 and Elp900, respectively. Nontransplantation was used as a negative control. To prevent immune rejection of xenogeneic mouse cells, the rats were subcutaneously injected daily with cyclosporine A (LC Laboratories, Woburn, MA, USA) [28]. At 3 weeks after implantation, the rats were sacrificed and calvariae were entirely extracted. The specimens were fixed with 10% neutral-buffered formalin solution (Wako Pure Chemical) overnight before microcomputed tomography (CT) analysis. Subsequently, the specimens were decalcified and embedded in paraffin, followed by cutting into sections at the center of the graft in the coronal plane. Sections were then analyzed via histological and immunofluorescent staining.

### 2.8. Micro-CT and Bone Morphometric Analyses

The bone mineral density (BMD) of newly formed bone was determined using a ScanXmate-E090 three-dimensional micro-X-ray CT imager (Comscan Tecno Co., Ltd., Kanagawa, Japan) and TRI/3D-BON bone structure analysis software (Ratoc System Engineering, Tokyo, Japan). Calvariae were X-rayed at an energy level of 68 kVp and a current of 100 mA through a 1 mm thick brass filter. The isotropic voxel size was 50.38 *μ*m/pixel, and 3D reconstruction was performed using the calibration curve of the bone mineral content obtained by scanning of a hydroxyapatite phantom under the same X-ray conditions. Round defect sites with a diameter of 5 mm were selected for analysis of the density of newly formed bone. The specific thresholds for bone tissue were determined by superimposing segmented images over the original grayscale X-ray images.

### 2.9. Statistical Analyses

One-way analysis of variance (ANOVA) with Tukey's or Dunnett's post hoc test was used for comparison of more than two groups. *P* < 0.05 was considered to be statistically significant.

## 3. Results

### 3.1. Effects of Microspace Size on Cell-Cell Contact and Compaction of iPSC Aggregates

After 2 days of aggregation, iPSC constructs of different sizes were observed in the Elp400, Elp500, and Elp900 groups: 200, 280, and 350 *μ*m, respectively. The morphology of cell aggregates was different among the three groups. Elp400 demonstrated the most uniformity with smooth-surfaced iPSC aggregates, whereas scalloped surfaces were observed in both Elp500 and Elp900 groups (Figure 1(a)). After RA treatment, no marked difference in iPSC aggregate size was observed in Elp400; in contrast, a significantly smaller size was observed in Elp500 and Elp900. The decrease in size observed in Elp500 was higher than that in Elp900 (Figure 1(b)). Before RA treatment, *E-cadherin* mRNA expression was higher in Elp400 and Elp500 than in Elp900. After RA treatment, *E-cadherin* mRNA expression was significantly decreased in all groups, with a significantly greater decrease in Elp900 than in Elp400 and Elp500 (Figure 1(c)). Hoechst staining showed dense nuclei in constructs fabricated using Elp400 and Elp500, whereas sparser nuclei were observed in the constructs fabricated using Elp900 (Figure 1(d)).

### 3.2. Effects of Microspace Size on Growth, Morphology, and Viability of OI-iPSC Constructs

**Figure 2:**
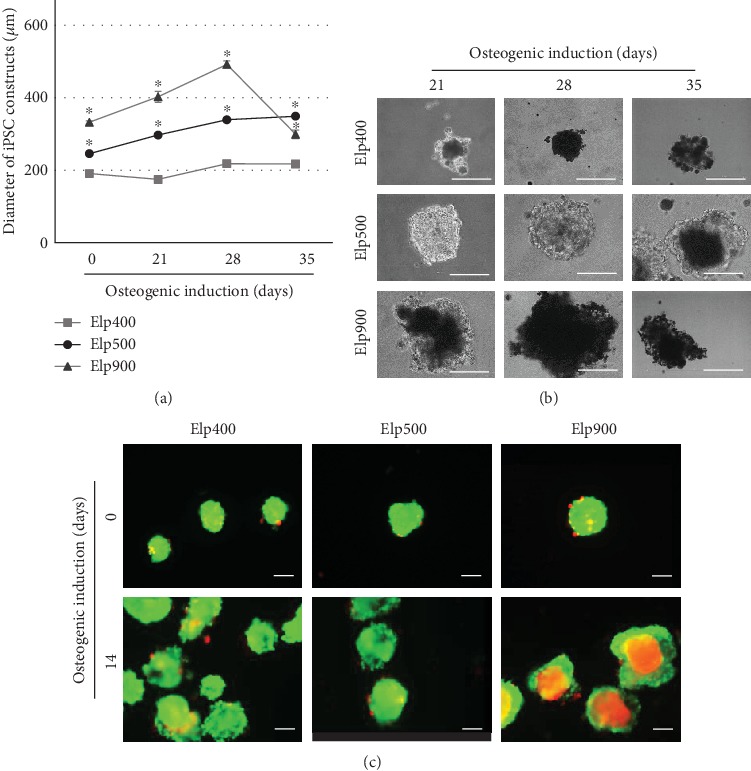
Microspace size influences growth rate and viability of OI-iPSC constructs during osteogenic differentiation. (a) Size and growth rate of iPSC constructs during osteogenic induction in different-sized microspaces at days 0, 21, 28, and 35. Asterisks indicate statistically significant difference for Elp500 and Elp900 compared to Elp400 (*P* < 0.05, ANOVA with Dunnett's multiple comparison test). The data represent the mean ± SD (*n* = 3). (b) Representative morphological images of OI-iPSC constructs cultured in Elp400, Elp500, and Elp900 at days 21, 28, and 35 after osteogenic initiation. Scale bars: 200 *μ*m. (c) Viability of cells in iPSC constructs before osteogenic induction (day 0) and 14 days after osteogenic commencement (day 14) was demonstrated using the Live/Dead Viability/Cytotoxicity Kit. Scale bars: 200 *μ*m.

The OI-iPSC construct growth rate in Elp400, Elp500, and Elp900 gradually increased from day 0 to day 28. OI-iPSC constructs cultured using Elp400 demonstrated the lowest growth rate, whereas the highest growth rate was observed in Elp900. The size of OI-iPSC constructs cultured for 35 days was decreased in Elp900, in contrast to Elp400 and Elp500. The largest OI-iPSC constructs were observed in Elp500 at day 35 (Figure 2(a)). On visual examination, 28- and 35-day OI-iPSC constructs in Elp400 and Elp900 became soft and fragile, especially in Elp900, where the constructs could not maintain their ball-like morphology and the size decreased from 500 to 300 *μ*m. In contrast, well-formed ball-like constructs were only found in the Elp500 group at all time points (Figure 2(b)). Homogeneous construct size was observed in Elp500, but not in Elp400 and Elp900. Central necrosis was only observed in Elp900 constructs, whereas Elp500 constructs had the smallest amount of dead cells (Figure 2(c)).

### 3.3. Effects of Microspace Size on Osteogenic Differentiation of OI-iPSC Constructs

#### 3.3.1. Expression of Osteogenic Marker Genes

**Figure 3:**
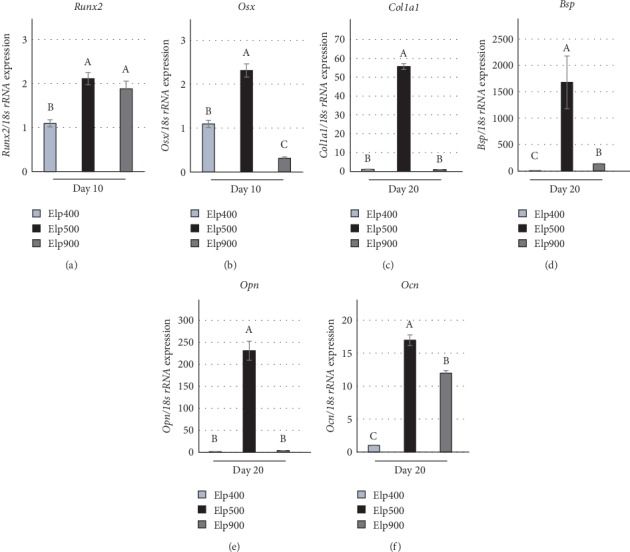
Microspace size influences the osteogenic marker gene expression of OI-iPSC constructs. The expression of osteogenic marker genes in Elp400, Elp500, and Elp900 was determined by real-time RT-PCR. (a and b) The gene expression of osteogenic-related transcriptional factors including (a) *Runx2* and (b) *Osterix* (*Osx*) was determined at day 10 of osteogenic induction. (c–f) After maintenance for 20 days, the expression of bone ECM-related genes-related genes such as (c) *Collagen 1a1* (*Col1a1*), (d) *Bone sialoprotein* (*Bsp*), (e) *Osteopontin* (*Opn*), and (f) *Osteocalcin* (*Ocn*) was evaluated. *18*s *rRNA* expression was used as an internal control. Different letters indicate significant differences between groups (*P* < 0.05, ANOVA with Tukey's multiple comparison test). The data represent the mean ± SD (*n* = 3).

After osteogenic cultivation for 10 days, *Runx2* gene expression was significantly higher in Elp500 and Elp900 constructs than in Elp400 constructs (Figure 3(a)). At the same time, *Osterix* (*Osx*) gene expression was higher in Elp500 constructs than in both Elp400 and Elp900 constructs (Figure 3(b)). Expression of bone ECM-related genes such as *Collagen 1a1* (*Col1a1*), *Bone sialoprotein* (*Bsp*), *Osteopontin* (*Opn*), and *Osteocalcin* (*Ocn*) was highest in the Elp500 group after 20 days (Figures 3(c)–3(f)).

#### 3.3.2. Histology and Mineralization of OI-iPSC Constructs

**Figure 4:**
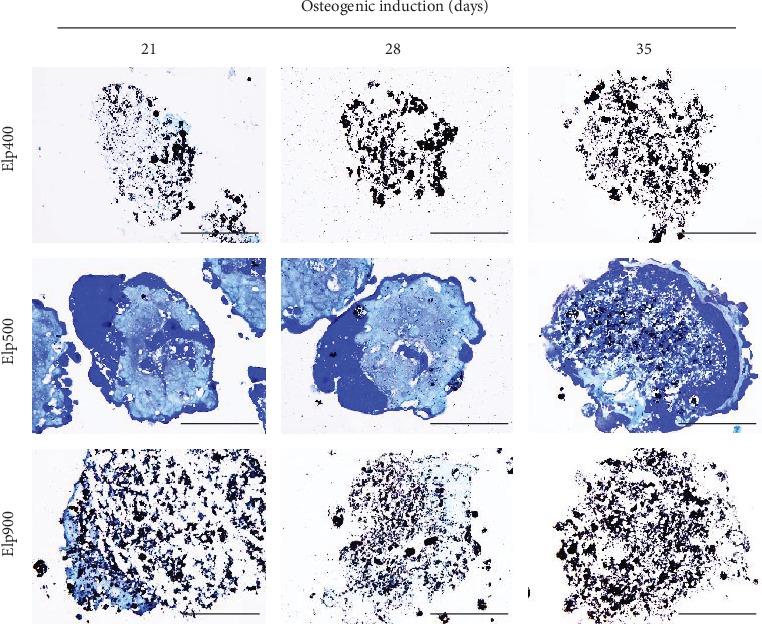
Microspace size influences the morphology and mineralization of OI-iPSC constructs. Representative images of histological analysis of OI-iPSC constructs in Elp400, Elp500, and Elp900. Paraffin-embedded sections (without decalcification) of OI-iPSC constructs at days 21, 28, and 35 were stained with von Kossa and counterstained with methylene blue. Black stain indicates mature mineralization. Dark blue and light blue indicate cells and ECM, respectively. Scale bars: 100 *μ*m.

High calcification was observed in OI-iPSC constructs fabricated using Elp400 and Elp900 at all time points, and only a small amount of ECM was detected (Figure 4). In contrast, OI-iPSC constructs cultured using Elp500 showed gradually increased mineralization from days 21, 28, and 35, and abundant ECM was observed at day 21. Sparse mineralization was detected in the ECM region at 28 days, whereas after 35 days, the constructs presented two layers of ECM: a homogeneous outer layer and a dense inner layer. To confirm the morphology of the OI-iPSC constructs, sequential sections were stained by HE (Supplementary Figure 2). Fragile and unstructured cell masses were observed in the Elp400 group, similar to the inner region of Elp900 constructs at all time points. In contrast, the rich ECM region of Elp500 constructs stained positive for eosin. Remarkably, after 35 days of osteogenic induction in Elp500, two ECM layers were confirmed with different intensities of eosin-stained color that were different from the cytoplasmic-stained color.

### 3.4. Effects of Microspace Size on Osteoid Formation of OI-iPSC Constructs

**Figure 5:**
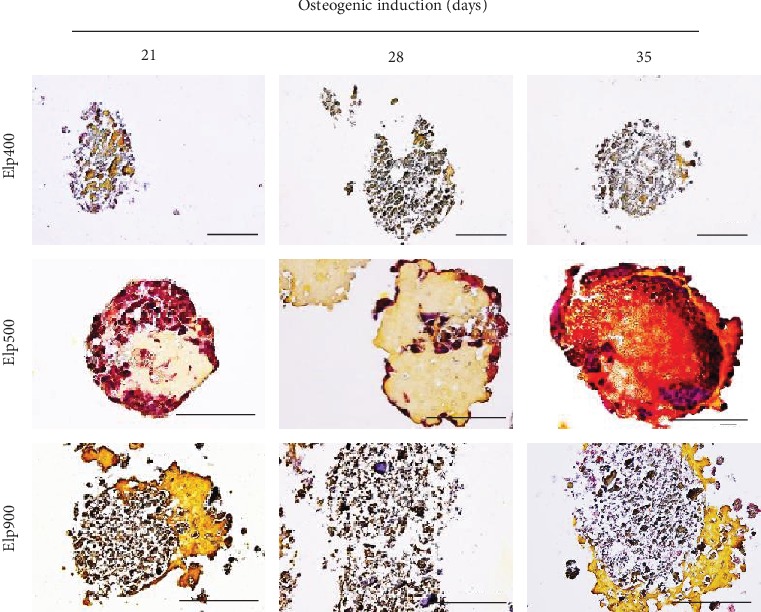
Microspace size influences osteoid formation of OI-iPSC constructs. Histological analysis using Movat's pentachrome staining was performed on paraffin-embedded sections (without decalcification) of OI-iPSC constructs at days 21, 28, and 35. Representative images of OI-iPSC constructs cultured in Elp400, Elp500, and Elp900 are shown. Different colors indicate different components of OI-iPSC constructs: black color for nuclei, red color for cytoplasm/osteoid/coarse collagen fibers, and yellow color for collagen fiber/calcification. Scale bars: 100 *μ*m.

Movat's pentachrome staining demonstrated fragile nuclei in 21, 28-, and 35-day OI-iPSC constructs cultured in Elp400, together with partly yellow staining of ECM and calcification (Figure 5). At the same time points, constructs fabricated using Elp900 demonstrated unstructured nuclear staining in the innermost area and homogeneously yellow staining of the surrounding ECM layer. In contrast, well-formed constructs were observed in Elp500 at all time points. The 21-day Elp500 OI-iPSC constructs showed formation of ECM. A larger ECM area was observed at day 28, together with the folding of osteogenic cells into the constructs, which then resulted in two cell layers: an outer and inner cell layer. After 35 days of osteogenic induction, red-stained osteoid tissue was observed mostly in the innerECM region, encapsulating the inner cell layer, and sparse yellow staining indicative of mineralization and/or collagen was observed in the inner region as well. The homogeneous outer ECM layer was stained yellow and also partly red.

### 3.5. Elp500 Accelerates Self-Organized Osteogenic Tissue Formation of OI-iPSC Constructs including Osteoblast-Like Cells, Osteoid/Osteogenic Tissue, and Mineralization

**Figure 6:**
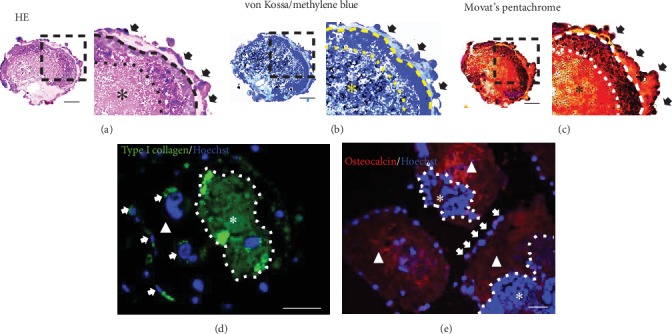
Identification of bone ECM related protein in OI-iPSC constructs cultured for 35 days in Elp500. Histological analysis was performed using HE, methylene blue-counterstained von Kossa, and Movat's pentachrome staining (without decalcification of sections). (a–c) Representative images of OI-iPSC constructs stained with (a) HE, (b) methylene blue-counterstained von Kossa, and (c) Movat's pentachrome. The right panels show magnifications of the dashed square regions. Black arrows indicate the outer cell layer. The inner cell layer is located in between the dashed line and dotted line. The inner ECM is indicated by asterisks. (d and e) Immunofluorescent assessment of (d) type I collagen and (e) osteocalcin in OI-iPSC constructs. The white dotted line separates OI-iPSC constructs into two layers; the outer layer is indicated by triangles and the inner layer is indicated by asterisks. Positive staining of cells for type I collagen and osteocalcin is demonstrated using white arrows. Scale bars: 50 *μ*m.

As shown in Figure 6(a), OI-iPSC constructs cultured for 35 days in Elp500 demonstrated two ECM layers, i.e., outer (the area between the outer cell layer and the dashed line) and inner (asterisk) layers, and two cellular regions, i.e., an outer region (black arrow) and an inner region (the area between the dashed and dotted lines). The outer ECM layer was surrounded by a single outer cellular region. Next to this layer, the inner cell region showed a dense cell mass and necrotic cell area indicated by disrupted nuclear staining. The innermost area located inside the dotted line in Figure 6(a) demonstrated a dense ECM layer. Moreover, sparse mineralization identified by von Kossa staining was observed mostly in the innermost ECM layer (Figure 6(b)). Movat's pentachrome staining confirmed osteoid formation in the innermost ECM area, which encapsulated the inner cell region. Sparse yellow staining was observed in the osteoid area of the innermost region (Figure 6(c)). Type I collagen was mainly observed in the inner ECM layer (asterisk) (Figure 6(d)). Osteocalcin was observed mainly in the outer ECM layer (triangles) (Figure 6(e)), and less type I collagen was detected in this area. Cells expressing type I collagen and osteocalcin were also predominantly identified in the outer layer of these constructs (arrows; Figures 6(d) and 6(e)).

### 3.6. OI-iPSC Constructs Fabricated from Elp500 Possess High Capacity to Form Bone Tissue in a Critical-Size Defect Model

**Figure 7:**
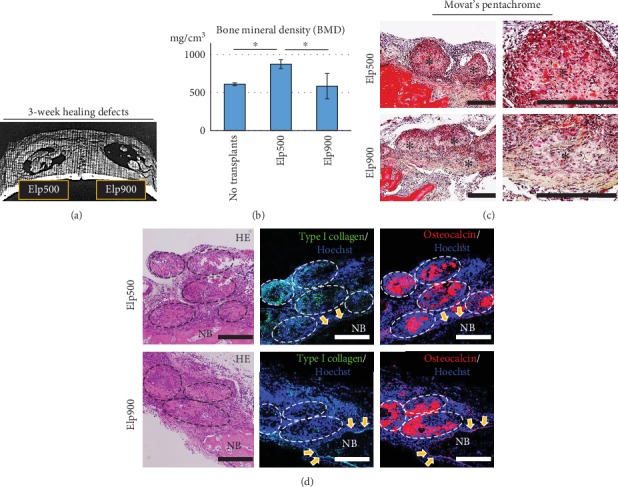
Comparative bone regeneration capacity of OI-iPSC constructs fabricated using Elp500 and Elp900 in critical-size calvarial defects. After healing for 3 weeks, new bone formation was evaluated using micro-CT and histological and immunofluorescent staining. (a) Representative 3D reconstruction image from micro-CT showing new bone formation in the defect area. (b) New bone formation was quantitatively analyzed by bone morphometry. Asterisks indicate statistically significant differences (*P* < 0.05, ANOVA with Tukey's multiple comparison test). The data represent the mean ± SD (*n* = 4). (c) Movat's pentachrome staining of decalcified sections of a defect after healing for 3 weeks. Right panels show magnifications of the OI-iPSC constructs of the left panels. Asterisks indicate the constructs. Scale bars: 200 *μ*m. (d) Immunofluorescent analysis of type I collagen and osteocalcin in remaining OI-iPSC constructs. The dashed oval regions show the remaining OI-iPSC constructs. Yellow arrows indicate osteoblasts lining the new bone (NB) area. Scale bars: 200 *μ*m.

After 3 weeks of healing, micro-CT imaging showed robust bone formation at the center of the defect site in the Elp500 group, whereas clusters of bone formation were sparsely observed in the defect area in the Elp900 group (Figure 7(a)). BMD was significantly higher in the Elp500 group than in the Elp900 group and nontransplanted group (Figure 7(b)). Movat's pentachrome showed positive red staining of bone tissue and osteoid tissue in decalcified sections. After 3 weeks of healing, OI-iPSC constructs were still observed in the transplanted area in both groups. Elp500 constructs showed red-stained tissues. In contrast, most of the staining in Elp900 was yellow, partially overlapped with red in the surrounding construct area (Figure 7(c)). Immunofluorescent analyses showed strong osteocalcin expression in both Elp500 and Elp900 groups. In contrast, type I collagen expression was higher in Elp500 than in Elp900 (Figure 7(d)).

## 4. Discussion

Organoids derived from self-organizing differentiation of iPSCs provide a unique system to examine mechanisms of organ development and disease, which could be useful for regenerative therapy and drug screening [29]. Understanding normal tissue organization may improve tissue engineering approaches because the regulation of developmental and regenerative processes of normal tissue shares common traits [30]. Although proper bone organoids have not yet been well established, Kale et al. described a promising approach to form spheroids of human adult bone precursor cells that requires 3D aggregation of the cells [31]. To mimic the developmental process under which mesenchymal condensation initiates bone formation, RA treatment has been used to guide iPSC constructs to differentiate toward an immature mesenchymal lineage before osteogenic initiation [32, 33]. In the present study, condensation of iPSC aggregates, indicated by a significant decrease in size, was observed in both Elp500 and Elp900, but not in Elp400 (Figure 1(b)), indicating that the microspace size influenced iPSC aggregation behavior during RA treatment.

E-cadherin is a master regulatory molecule for cell-cell adhesion in stem cells including pluripotent stem cells and MSCs [34, 35]. A previous report demonstrated that *E-cadherin* expression in EBs formed in smaller microspaces is higher than that in EBs formed in larger microspaces after maintenance in growth medium for 10 days [35]. Moreover, in 3D MSC culture, smaller cell constructs were associated with *E-cadherin* expression [34]. These previous findings could support our finding of the highest *E-cadherin* expression in Elp400, together with a smooth surface indicating saturated condensation by day 2; therefore, no more condensation was observed at day 5. In contrast, *E-cadherin* expression of iPSC aggregates in Elp500 was not significantly different from that in Elp400, but significantly higher than that in Elp900. These results indicate that iPSC aggregates generated using Elp500 possessed stronger cell-cell adhesion potential than those generated using Elp900, as confirmed by tightly aggregated Hoechst-stained nuclei. These findings indicate that microspace size could regulate, at least in part, the behavior of iPSC aggregates prior to osteogenic initiation via E-cadherin.

The fabrication of 3D tissue/organoids requires suitable microplatforms that can maintain cell viability for long-term culture [36]. In the present study, the Elp500 group demonstrated high cell viability and well-formed ball-like constructs, whereas fragile and soft constructs were observed in the Elp400 and Elp900 groups. Pettinato et al. reported that EB size affected viability [37]. Van Winkle et al. showed that large EBs (diameter of 800 *μ*m) underwent core necrosis because the oxygen concentration at the center was 50% lower than that in smaller EBs (diameter of 400 *μ*m) [38]. It is thus possible that the large constructs generated using Elp900 underwent partial/central necrosis, resulting in a size decrease at day 28. Although the Elp400 constructs were the smallest, their fragility might also indicate necrosis. In a previous study [35], higher glucose consumption and lactate production were observed in EBs cultured in 400 *μ*m microspaces than in those cultured in 600 *μ*m microspaces. This may have happened because of the microspace structure and/or formation of dense EBs that reduced the diffusion of molecules necessary for cell survival, such as oxygen, nutrients, and secreted molecules. This finding indicates that 400 *μ*m microspaces induced anaerobic metabolism through the generation of an inappropriate environment, leading to difficulty in maintaining cell viability for long-term culture [35]. These findings support our interpretation that Elp400 might not provide a proper microenvironment to maintain OI-iPSC construct viability in long-term culture.

Runx2 and Osterix are master transcriptional regulators of early osteogenic differentiation in several types of cells [39, 40]. Runx2 expression has been found in nonskeletal tissue including sperm and brain [41]. In contrast, *Osx* knockout mice show complete abrogation of bone formation, suggesting that Osterix is a specific transcriptional factor for osteogenic differentiation [42]. These characteristics suggest steady osteogenesis in our study, as *Runx2* and *Osx* were most highly expressed in Elp500. Bone extracellular matrix-related genes, such as *Col1a1*, *Opn*, *Bsp*, and *Ocn*, are regulated by downstream transcriptional activation of Runx2 and Osterix [43]. As we expected, the highest mRNA expression of these genes was observed in Elp500. Therefore, these results suggest that Elp500 provides optimal conditions for differentiation of iPSCs toward osteogenic lineages to form osteogenic tissue *in vitro*.

Both bone mineralization (amorphous calcium-phosphate accumulation and carbonated hydroxyapatite crystal formation) and calcification (calcium salt deposition) are detected by von Kossa staining [44, 45]. OI-iPSC constructs in the Elp400 and Elp900 groups demonstrated strongly von Kossa staining in the same areas where fragile nuclei and nonstructured cell constructs were observed. Previous studies reported that necrotic cells serve as nuclei for calcium-phosphate deposition, which usually occurs as ectopic calcification [46–48]. The positive von Kossa staining observed here might indicate calcification in the necrotic area. In contrast, positive von Kossa staining was observed in the ECM area of OI-iPSC constructs fabricated using Elp500, together with osteogenic cells as indicated by high expression of osteogenic proteins suggestive of bone mineral deposits. Although von Kossa staining alone might not be sufficient to confirm that mineralization *in vitro* represents bone formation [49], our group has previously demonstrated hydroxyapatite in the calcified zone in the ECM of iPSC constructs using selected area election diffraction (SAED) [11].

Physiological formation of mineralized bone occurs through mineral deposition in osteoid tissue, which mainly consists of type I collagen [50, 51]. We observed osteoid formation during osteogenic induction and only found osteoid tissue at day 35 in the constructs fabricated using Elp500. This osteoid tissue was mainly detected in the inner ECM area together with robust expression of type I collagen and osteocalcin. In addition, von Kossa staining confirmed mature mineralization in the inner ECM region. These findings suggest generation of osteoid-derived biomimetic bone in the Elp500 group. *In vivo* data showed osteoid staining, with type I collagen expression in the ECM and surrounding area of remaining OI-iPSC constructs. Stronger expression was observed in the Elp500 group than in the Elp900 group, confirming the generation of osteoid-rich constructs in the Elp500 group. The OI-iPSC constructs in the transplanted area demonstrated strong osteocalcin expression in both groups, which corresponded to the observed *in vitro Ocn* gene expression. In addition, 35-day Elp500 constructs showed strong osteocalcin expression in the ECM and osteogenic cells in the outer layer. Collagenous proteins including type I collagen and noncollagenous proteins including osteocalcin are major components of bone tissue [52, 53]. The coexpression of type I collagen and osteocalcin in OI-iPSC constructs suggests that Elp500 is a promising tool to guide the differentiation of iPSCs toward osteogenic lineages and promote self-organizing osteogenic tissue formation *in vitro*.

Biomaterials for bone regeneration can be classified as osteogenic, osteoinductive, and osteoconductive. Osteoinduction by transplant materials is crucial for effective bone regeneration in critical-size defects [4, 54]. In the present study, before transplantation, the constructs of both groups showed an outer ECM layer. However, the inner layer was different: abundant osteoids in Elp500 and calcification of necrotic cells in Elp900 (Figure 5). In addition, osteogenic marker gene expression suggested more mature osteogenic cells in Elp500 constructs than in Elp900 constructs, implying higher osteoinductivity in Elp500 constructs. Indeed, after 3 weeks of healing, the amount of new bone formation was higher in the Elp500 group than in the Elp900 group. Interestingly, the new bone formation in both groups was observed at the center of the defect site, which was different from usual healing that occurs from the defect border when using other materials such as beta tricalcium phosphate (*β*-TCP) and inorganic bovine bone (Bio-Oss) [55]. These suggest that OI-iPSC constructs possessed osteoinductive characteristics, and that OI-iPSC constructs fabricated and maintained in Elp500 were superior. We further confirmed the enhanced bone formation in Elp500 through comparison to the nontransplanted group. Significantly increased new bone formation was observed in the Elp500 group, suggesting a high capacity for *in vivo* bone regeneration. Although OI-iPSC constructs prepared in Elp900 had osteoinductive properties, they did not show enhanced bone formation *in vivo*. This lack of *in vivo* efficacy might have occurred through inflammatory responses to cell death/debris in the constructs that impair bone healing [56].

As described above, in this study, we transplanted living OI-iPSC constructs and observed their bone regeneration capacity in critical-size defects to study the effects of microspace size on osteogenic differentiation of iPSC constructs. The enhanced bone formation in the Elp500 group was expected to be modulated by iPSC-derived osteogenic cells and osteoid constructs. Our results (Supplementary Figures 3A and 3B) are also supported by the fact that osteogenic cells secrete factors to control osteogenic differentiation of MSCs [57], and thus the constructs might have also had stimulatory effects on endogenous cells. For therapeutic application as osteoinductive graft materials, we also demonstrated that freeze-dried OI-iPSC constructs promote adhesion and osteogenic differentiation of MSCs (Supplementary Figures 3C and 3D). These data confirmed that microspace size affected the osteogenic differentiation of iPSC constructs, and that OI-iPSC constructs fabricated using the optimal microspace size might be a good graft material/scaffold for improving bone regeneration Despite the lack of teratoma formation here, further studies are necessary to confirm the safety of cell-containing constructs.

Our results suggest that effective fabrication of biomimetic bone requires an optimal microspace size to orchestrate early cell condensation, mimicking mesenchymal condensation, and to allow self-organizing osteogenic differentiation of iPSC constructs. OI-iPSC constructs fabricated using Elp500 demonstrated a heterogeneous population of bone progenitors, including osteoblasts that could form osteoid tissue, followed by mineralization to generate biomimetic bone. Bone is a mineralized connective tissue and a highly dynamic organ that is continuously resorbed by osteoclasts and formed by osteoblasts, whereas osteocytes act as mechanosensors and orchestrators of the remodeling process [58]. This complexity necessitates further studies to achieve the fabrication of proper bone organoids. Although the present study demonstrated the effects of microspace size on osteogenic differentiation of iPSC constructs, which focuses only on the aspect of bone formation, our findings could represent a key step for the development of bioengineered bone organoids for regenerative therapy, disease modeling, and drug screening.

## 5. Conclusion

This study demonstrated that microspace size, which is capable of providing different stem cell niches, affects iPSC behaviors and osteogenic commitment. A specific microspace diameter of 500 *μ*m (Elp500) provided and maintained a suitable microenvironment for self-organizing osteogenic commitment of iPSCs to form osteoid-rich constructs. These constructs possessed high bone regeneration capacity for *in vivo* bone healing of critical-size defects. Thus, microspace culture using Elp500 could represent a microplatform for development of organoid technologies for bone regeneration.

## Data Availability

We state that where data supporting the results reported in a published article can be found, including, where applicable, hyperlinks to publicly archived data sets analyzed or generated during the study.

## Supplementary Materials

Supplementary Materials Supplementary Materials and Methods: osteoinductivity assessment of OI-iPSC constructs. Supplementary Table 1: primers used for SYBR Green real-time RT-PCR. Supplementary Figure 1: homogeneous formation of iPSC aggregates in different microspace sizes after seeding for 0, 6, 12, 24, and 48 hours. iPSC aggregates were observed under a phase contrast microscope. Representative images show single iPSCs at the bottom of a microspace well at 0 and 6 hours. The aggregation of iPSCs was observed for 12 hours. After 24 hours, different sizes of iPSC aggregates were observed in the different microspace sizes of Elp400, Elp500, and Elp900. Scale bars: 200 *μ*m. Supplementary Figure 2: microspace size influences the OI-iPSC constructs' morphology. The OI-iPSC constructs were subjected to osteogenic induction for 21, 28, and 35 days. Histological analysis was performed on paraffin-embedded sections (without decalcification) via HE staining. Scale bars: 100 *μ*m. Supplementary Figure 3: osteoinductive properties of OI-iPSC constructs fabricated using Elp500 (see “Supplementary Materials and Methods” for details). (A and B) Effects of conditioned medium (CM) of 35-day OI-iPSC constructs on LT-MSC proliferation and osteogenic differentiation. Scale bars: 200 *μ*m. (A) Methylene blue and (B) von Kossa staining of 14-day cultures. Arrows indicate mineral nodules positive for von Kossa staining. (C and D) LT-MSCs were cultured with freeze-dried OI-iPSC constructs in osteogenic medium for 14 days. Scale bars: 200 *μ*m (C) Representative images were obtained under phrase-contrast microscopy at days 0, 3, and 10 of osteogenic induction. The right panels show magnified images. Cells adhering to freeze-dried constructs are demonstrated by arrows. (D) Determination of mineralized deposits by histological analysis; 14-day cultures were stained with Alizarin Red S and von Kossa. Asterisks show areas positive for Alizarin Red S staining surrounding the freeze-dried OI-iPSC constructs. High-magnification images in the right panels demonstrate mineralized nodules. For von Kossa staining, arrows identify black-stained mineral deposit areas surrounding the constructs.

## References

[1] Egusa H., Sonoyama W., Nishimura M., Atsuta I., Akiyama K. (2012). Stem cells in dentistry—part I: stem cell sources. *Journal of Prosthodontic Research*.

[2] Yamada M., Egusa H. (2018). Current bone substitutes for implant dentistry. *Journal of Prosthodontic Research*.

[3] Amini A. R., Laurencin C. T., Nukavarapu S. P. (2012). Bone tissue engineering: recent advances and challenges. *Critical Reviews in Biomedical Engineering*.

[4] Egusa H., Sonoyama W., Nishimura M., Atsuta I., Akiyama K. (2012). Stem cells in dentistry—part II: clinical applications. *Journal of Prosthodontic Research*.

[5] O'Brien F. J. (2011). Biomaterials & scaffolds for tissue engineering. *Materials Today*.

[6] Watanabe J., Yamada M., Niibe K. (2018). Preconditioning of bone marrow-derived mesenchymal stem cells with N-acetyl-L-cysteine enhances bone regeneration via reinforced resistance to oxidative stress. *Biomaterials*.

[7] Egusa H., Okita K., Kayashima H. (2010). Gingival fibroblasts as a promising source of induced pluripotent stem cells. *PLoS One*.

[8] Egusa H., Kayashima H., Miura J. (2014). Comparative analysis of mouse-induced pluripotent stem cells and mesenchymal stem cells during osteogenic differentiation in vitro. *Stem Cells and Development*.

[9] Kanke K., Masaki H., Saito T. (2014). Stepwise differentiation of pluripotent stem cells into osteoblasts using four small molecules under serum-free and feeder-free conditions. *Stem Cell Reports*.

[10] Jeon O. H., Panicker L. M., Lu Q., Chae J. J., Feldman R. A., Elisseeff J. H. (2016). Human iPSC-derived osteoblasts and osteoclasts together promote bone regeneration in 3D biomaterials. *Scientific Reports*.

[11] Okawa H., Kayashima H., Sasaki J. I. (2016). Scaffold-free fabrication of osteoinductive cellular constructs using mouse gingiva-derived induced pluripotent stem cells. *Stem Cells International*.

[12] Park D., Lim J., Park J. Y., Lee S. H. (2015). Concise review: stem cell microenvironment on a chip: current technologies for tissue engineering and stem cell biology. *Stem Cells Translational Medicine*.

[13] Lu Z., Roohani-Esfahani S. I., Wang G., Zreiqat H. (2012). Bone biomimetic microenvironment induces osteogenic differentiation of adipose tissue-derived mesenchymal stem cells. *Nanomedicine*.

[14] Tong W., Brown S. E., Krebsbach P. H. (2007). Human embryonic stem cells undergo osteogenic differentiation in human bone marrow stromal cell microenvironments. *Journal of Stem Cells*.

[15] Zheng G., Xie Z., Wang P. (2019). Enhanced osteogenic differentiation of mesenchymal stem cells in ankylosing spondylitis: a study based on a three-dimensional biomimetic environment. *Cell Death & Disease*.

[16] Kim J., Ma T. (2012). Perfusion regulation of hMSC microenvironment and osteogenic differentiation in 3D scaffold. *Biotechnology and Bioengineering*.

[17] Hsiao C., Tomai M., Glynn J., Palecek S. P. (2014). Effects of 3D microwell culture on initial fate specification in human embryonic stem cells. *AICHE Journal*.

[18] Xie A. W., Binder B. Y. K., Khalil A. S. (2017). Controlled self-assembly of stem cell aggregates instructs pluripotency and lineage bias. *Scientific Reports*.

[19] Limraksasin P., Kondo T., Zhang M. (2020). In vitro fabrication of hybrid bone/cartilage complex using mouse induced pluripotent stem cells. *International Journal of Molecular Sciences*.

[20] Hsiao C., Palecek S. P. (2012). Microwell regulation of pluripotent stem cell self-renewal and differentiation. *Bionanoscience*.

[21] Takebe T., Sekine K., Kimura M. (2017). Massive and reproducible production of liver buds entirely from human pluripotent stem cells. *Cell Reports*.

[22] Miyamoto D., Nakazawa K. (2016). Differentiation of mouse iPS cells is dependent on embryoid body size in microwell chip culture. *Journal of Bioscience and Bioengineering*.

[23] Hwang Y. S., Chung B. G., Ortmann D., Hattori N., Moeller H. C., Khademhosseini A. (2009). Microwell-mediated control of embryoid body size regulates embryonic stem cell fate via differential expression of WNT5a and WNT11. *Proceedings of the National Academy of Sciences*.

[24] Movat H. Z. (1955). Demonstration of all connective tissue elements in a single section-pentachrome stains. *Archives of Pathology*.

[25] Russell H. K. (1972). Modification of Movats pentachrome stain. *Archives of Pathology*.

[26] Olah A. J., Simon A., Gaudy M., Herrmann W., Schenk R. K. (1977). Differential staining of calcified tissues in plastic embedded microtome sections by a modification of Movats pentachrome stain. *Stain Technology*.

[27] Egusa H., Kaneda Y., Akashi Y. (2009). Enhanced bone regeneration via multimodal actions of synthetic peptide SVVYGLR on osteoprogenitors and osteoclasts. *Biomaterials*.

[28] Toh W. S., Lee E. H., Guo X. M. (2010). Cartilage repair using hyaluronan hydrogel-encapsulated human embryonic stem cell-derived chondrogenic cells. *Biomaterials*.

[29] Lancaster M. A., Huch M. (2019). Disease modelling in human organoids. *Disease Models & Mechanisms*.

[30] Grassi L., Alfonsi R., Francescangeli F. (2019). Organoids as a new model for improving regenerative medicine and cancer personalized therapy in renal diseases. *Cell Death & Disease*.

[31] Kale S., Biermann S., Edwards C., Tarnowski C., Morris M., Long M. W. (2000). Three-dimensional cellular development is essential for ex vivo formation of human bone. *Nature Biotechnology*.

[32] Bilousova G., Jun D. H., King K. B. (2011). Osteoblasts derived from induced pluripotent stem cells form calcified structures in scaffolds both in vitro and in vivo. *Stem Cells*.

[33] Tashiro K., Inamura M., Kawabata K. (2009). Efficient adipocyte and osteoblast differentiation from mouse induced pluripotent stem cells by adenoviral transduction. *Stem Cells*.

[34] Lee Y. B., Kim E. M., Byun H. (2018). Engineering spheroids potentiating cell-cell and cell-ECM interactions by self-assembly of stem cell microlayer. *Biomaterials*.

[35] Nakazawa K., Yoshiura Y., Koga H., Sakai Y. (2013). Characterization of mouse embryoid bodies cultured on microwell chips with different well sizes. *Journal of Bioscience and Bioengineering*.

[36] Sun H., Jia Y., Dong H., Dong D., Zheng J. (2020). Combining additive manufacturing with microfluidics: an emerging method for developing novel organs-on-chips. *Current Opinion in Chemical Engineering*.

[37] Pettinato G., Wen X., Zhang N. (2015). Engineering strategies for the formation of embryoid bodies from human pluripotent stem cells. *Stem Cells and Development*.

[38] Van Winkle A. P., Gates I. D., Kallos M. S. (2012). Mass transfer limitations in embryoid bodies during human embryonic stem cell differentiation. *Cells, Tissues, Organs*.

[39] Zhang C. (2010). Transcriptional regulation of bone formation by the osteoblast-specific transcription factor Osx. *Journal of Orthopaedic Surgery and Research*.

[40] James A. W. (2013). Review of signaling pathways governing MSC osteogenic and adipogenic differentiation. *Scientifica*.

[41] Jeong J. H., Jin J. S., Kim H. N. (2008). Expression of Runx 2 transcription factor in non-skeletal tissues, sperm and brain. *Journal of Cellular Physiology*.

[42] Zhou X., Zhang Z. P., Feng J. Q. (2010). Multiple functions of Osterix are required for bone growth and homeostasis in postnatal mice. *Proceedings of the National Academy of Sciences*.

[43] Chen Q., Shou P., Zheng C. (2016). Fate decision of mesenchymal stem cells: adipocytes or osteoblasts?. *Cell Death and Differentiation*.

[44] Wang Y. H., Liu Y., Maye P., Rowe D. W. (2006). Examination of mineralized nodule formation in living osteoblastic cultures using fluorescent dyes. *Biotechnology Progress*.

[45] Mechiche Alami S., Gangloff S. C., Laurent-Maquin D., Wang Y., Kerdjoudj H. (2016). Concise review: in vitro formation of bone-like nodules sheds light on the application of stem cells for bone regeneration. *Stem Cells Translational Medicine*.

[46] Tzimas G. N., Afshar M., Emadali A., Chevet E., Vali H., Metrakos P. P. (2004). Correlation of cell necrosis and tissue calcification with ischemia/reperfusion injury after liver transplantation. *Transplantation Proceedings*.

[47] Valdivielso J. M. (2011). Vascular calcification: types and mechanisms. *Nefrología*.

[48] Fujita H., Yamamoto M., Ogino T. (2014). Necrotic and apoptotic cells serve as nuclei for calcification on osteoblastic differentiation of human mesenchymal stem cells in vitro. *Cell Biochemistry and Function*.

[49] Bonewald L. F., Harris S. E., Rosser J. (2003). von Kossa staining alone is not sufficient to confirm that mineralization in vitro represents bone formation. *Calcified Tissue International*.

[50] Murshed M. (2018). Mechanism of bone mineralization. *Cold Spring Harbor Perspectives in Medicine*.

[51] Alford A. I., Kozloff K. M., Hankenson K. D. (2015). Extracellular matrix networks in bone remodeling. *The International Journal of Biochemistry & Cell Biology*.

[52] Carvalho M. S., Poundarik A. A., Cabral J. M. S., da Silva C. L., Vashishth D. (2018). Biomimetic matrices for rapidly forming mineralized bone tissue based on stem cell-mediated osteogenesis. *Scientific Reports*.

[53] Sroga G. E., Karim L., Colon W., Vashishth D. (2011). Biochemical characterization of major bone-matrix proteins using nanoscale-size bone samples and proteomics methodology. *Molecular & Cellular Proteomics*.

[54] Habibovic P., de Groot K. (2007). Osteoinductive biomaterials—properties and relevance in bone repair. *Journal of Tissue Engineering and Regenerative Medicine*.

[55] de Freitas S. L., de Carvalho Reis E. N. R., Barbara T. A. (2017). Assessment of bone repair in critical-size defect in the calvarium of rats after the implantation of tricalcium phosphate beta (*β*-TCP). *Acta Histochemica*.

[56] Rock K. L., Kono H. (2008). The inflammatory response to cell death. *Annual Review of Pathology*.

[57] Birmingham E., Department of Mechanical and Biomedical Engineering, National University of Ireland Galway, Galway, Ireland, Niebur G. L. (2012). Osteogenic differentiation of mesenchymal stem cells is regulated by osteocyte and osteoblast cells in a simplified bone niche. *European Cells and Materials*.

[58] Florencio-Silva R., Sasso G. R., Sasso-Cerri E., Simoes M. J., Cerri P. S. (2015). Biology of bone tissue: structure, function, and factors that influence bone cells. *BioMed Research International*.

