# Supplementary material for: Size-Optimized Microspace Culture Facilitates Differentiation of Mouse Induced Pluripotent Stem Cells into Osteoid-Rich Bone Constructs
Source: Stem Cells Int. 2020 May 14;2020:7082679. doi: 10.1155/2020/7082679 (PMC7244985; doi:10.1155/2020/7082679)
Supplement: Supplementary Materials — and Methods: osteoinductivity assessment of OI-iPSC constructs. Supplementary Table 1: primers used for SYBR Green real-time RT-PCR. Supplementary Figure 1: homogeneous formation of iPSC aggregates in different microspace sizes after seeding for 0, 6, 12, 24, and 48 hours. iPSC aggregates were observed under a phase contrast microscope. Representative images show single iPSCs at the bottom of a microspace well at 0 and 6 hours. The aggregation of iPSCs was observed for 12 hours. After 24 hours, different sizes of iPSC aggregates were observed in the different microspace sizes of Elp400, Elp500, and Elp900. Scale bars: 200 μm. Supplementary Figure 2: microspace size influences the OI-iPSC constructs' morphology. The OI-iPSC constructs were subjected to osteogenic induction for 21, 28, and 35 days. Histological analysis was performed on paraffin-embedded sections (without decalcification) via HE staining. Scale bars: 100 μm. Supplementary Figure 3: osteoinductive properties of OI-iPSC constructs fabricated using Elp500 (see “Supplementary Materials and Methods” for details). (A and B) Effects of conditioned medium (CM) of 35-day OI-iPSC constructs on LT-MSC proliferation and osteogenic differentiation. Scale bars: 200 μm. (A) Methylene blue and (B) von Kossa staining of 14-day cultures. Arrows indicate mineral nodules positive for von Kossa staining. (C and D) LT-MSCs were cultured with freeze-dried OI-iPSC constructs in osteogenic medium for 14 days. Scale bars: 200 μm (C) Representative images were obtained under phrase-contrast microscopy at days 0, 3, and 10 of osteogenic induction. The right panels show magnified images. Cells adhering to freeze-dried constructs are demonstrated by arrows. (D) Determination of mineralized deposits by histological analysis; 14-day cultures were stained with Alizarin Red S and von Kossa. Asterisks show areas positive for Alizarin Red S staining surrounding the freeze-dried OI-iPSC constructs. High-magnificat [file 7082679.f1.pdf]

Supplementary Materials and Methods (see "Supplementary Figure 3" for results)

1. Osteoinductivity assessment of OI-iPSC constructs

1.1. Osteoinductivity assessment of secreted factors from OI-iPSC constructs

Human bone marrow-derived MSCs sorted by LNGFR$^+$/THY-1$^+$ (LT-MSCs) [Mabuchi Y. *et al.*, *Stem Cell Reports*, 1: 152-65, 2013] were kindly provided by Dr. Y Mabuchi (Tokyo Medical and Dental University, Tokyo, Japan). LT-MSCs were cultured in growth medium (GM) containing Dulbecco's modified Eagle's medium (DMEM with 4.5 g/L glucose and without sodium pyruvate; Nacalai Tesque), 20% FBS (Hyclone), 1% penicillin-streptomycin (Wako Pure Chemical), 10 mM HEPES (Dojindo), and 10 ng/mL recombinant human basic fibroblast growth factor (bFGF; Wako Pure Chemical). LT-MSCs were maintained in GM until the cells reached 80% confluence.

Conditioned medium (CM) was collected from OI-iPSC constructs in the Elp500 condition at day 35. Then, LT-MSCs were prepared in 24-well plates and maintained in GM or OS medium with and without 10 μL/mL CM for 14 days. Half medium change was performed every 3 days. Subsequently, the cells were fixed with 10% neutral-buffered formalin solution (Wako Pure Chemical) prior to staining with von Kossa and methylene blue.

1.2. Osteoinductivity assessment of inactivated (freeze-dried) OI-iPSC constructs

OI-iPSC constructs were collected from Elp500 wells at day 35 and washed twice with PBS (Wako). Next, the constructs were pre-frozen at -80 °C overnight and subsequently transferred to a freeze-drying device (VD-250R; Taitec, Koshigaya, Japan) to inactivate the cells in the constructs at -10 °C under -0.1 MPa overnight.

LT-MSCs (2 x 10^4 cells/cm^2) were plated in 6-cm dishes containing freeze-dried OI-iPSC constructs from the Elp500 condition and maintained for 1 day. Next, GM was replaced by osteogenic medium (Zen-Bio, NC, USA) and the culture was maintained for 14 days. Half medium change was performed every 3 days. Then, the cells were fixed in 10% neutral-buffered formalin solution (Wako Pure Chemical) and subsequently stained using Alizarin Red S and von Kossa to detect mineralization nodules.

Supplementary Table 1: Primers used for SYBR Green real-time RT-PCR.

Description (gene name)	Primers (Fw, forward; Rv, reverse)	Product size (bp)	Accession number (reference)
E-cadherin (*CDH1*)	Fw: 5'-GTCTCCTCATGGCTTTGC-3'<br>Rv: 5'-CTTTAGATGCCGCTTCAC-3'	228	NM_009864.3 (Xiang X. et al., *PLoS One*, 7: e50781, 2012)
Runx2 (*Runx2*)	Fw: 5'-CGGGCTACCTGCCATCAC-3'<br>Rv: 5'-GGCCAGAGGCAGAAGTCAGA-3'	78	NM_001146038.2 (Speer M. Y. et al., *J Cell Biochem*, 110: 935–947, 2010)
Osterix (*Sp7*)	Fw: 5'-CTCGTCTGACTGCCTGCCTAG-3'<br>Rv: 5'-GCGTGGATGCCTGCCTTGTA-3'	84	NM_130458.3 (Fowlkes J. L. et al., *Endocrinology*, 149: 1697–1704, 2008)
Collagen 1a1 (*Col1a1*)	Fw: 5'-TGTCCCAACCCCCAAAGAC-3'<br>Rv: 5'-CCCTCGACTCCTACATCTTCTGA-3'	92	NM_007742.3 (Kaback L. A. et al., *J Cell Biochem*, 105: 219–226, 2008)
Osteocalcin (*Bglap*)	Fw: 5'-CCGGGAGCAGTGTGAGCTTA-3'<br>Rv: 5'-AGGCGGTCTTCAAGCCATACT-3'	68	NM_007541.3 (Jadlowiec J. et al., *J Biol Chem*, 279: 53323–53330, 2004)
Osteopontin (*Spp1*)	Fw: 5'-TCTCCTTGCGCCACAGAATG-3'<br>Rv: 5'-TCCTTAGACTCACCGCTCTT-3'	399	NM_001204201.1 (Yang J. et al., *Int J Mol Med*, 39: 1605-1612, 2017)
Bone sialoprotein (*Ibsp*)	Fw: 5'-CGGAGGAGACAACGGAGAAG-3'<br>Rv: 5'-GTAAGTGTCGCCACGAGGCT-3'	295	NM_ 008318.3 (Bouet G. et al., *PLoS One*, 10: e0117402, 2015)
18s rRNA	Fw: 5'-GTAACCCGTTGAACCCCATT-3'<br>Rv: 5'-CCATCCAATCGGTAGTAGCG-3'	151	X00686 (Valamehr B. et al., *Proc Natl Acad Sci U S A.*, 105: 14459-14464, 2008)

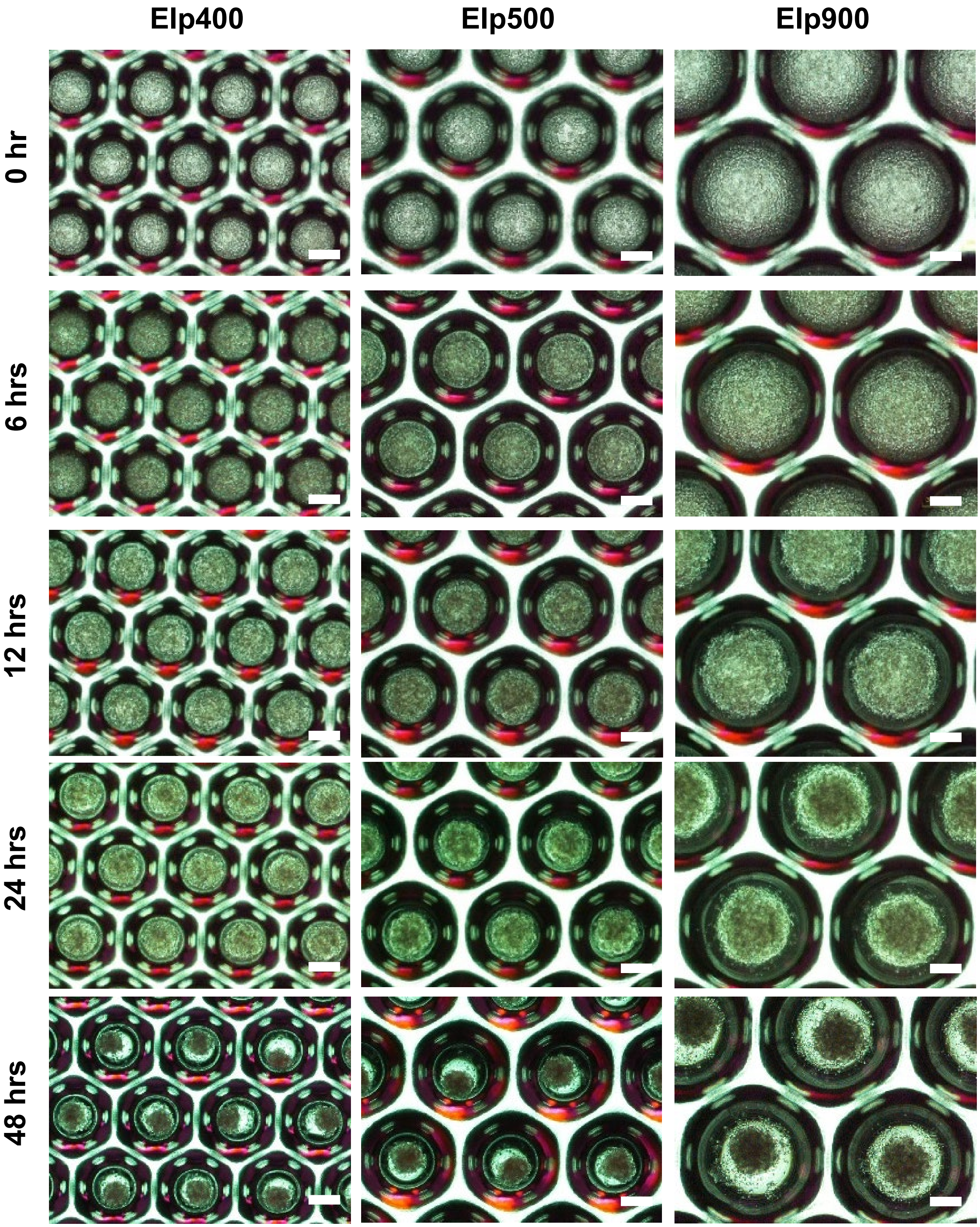

Supp Figure 1

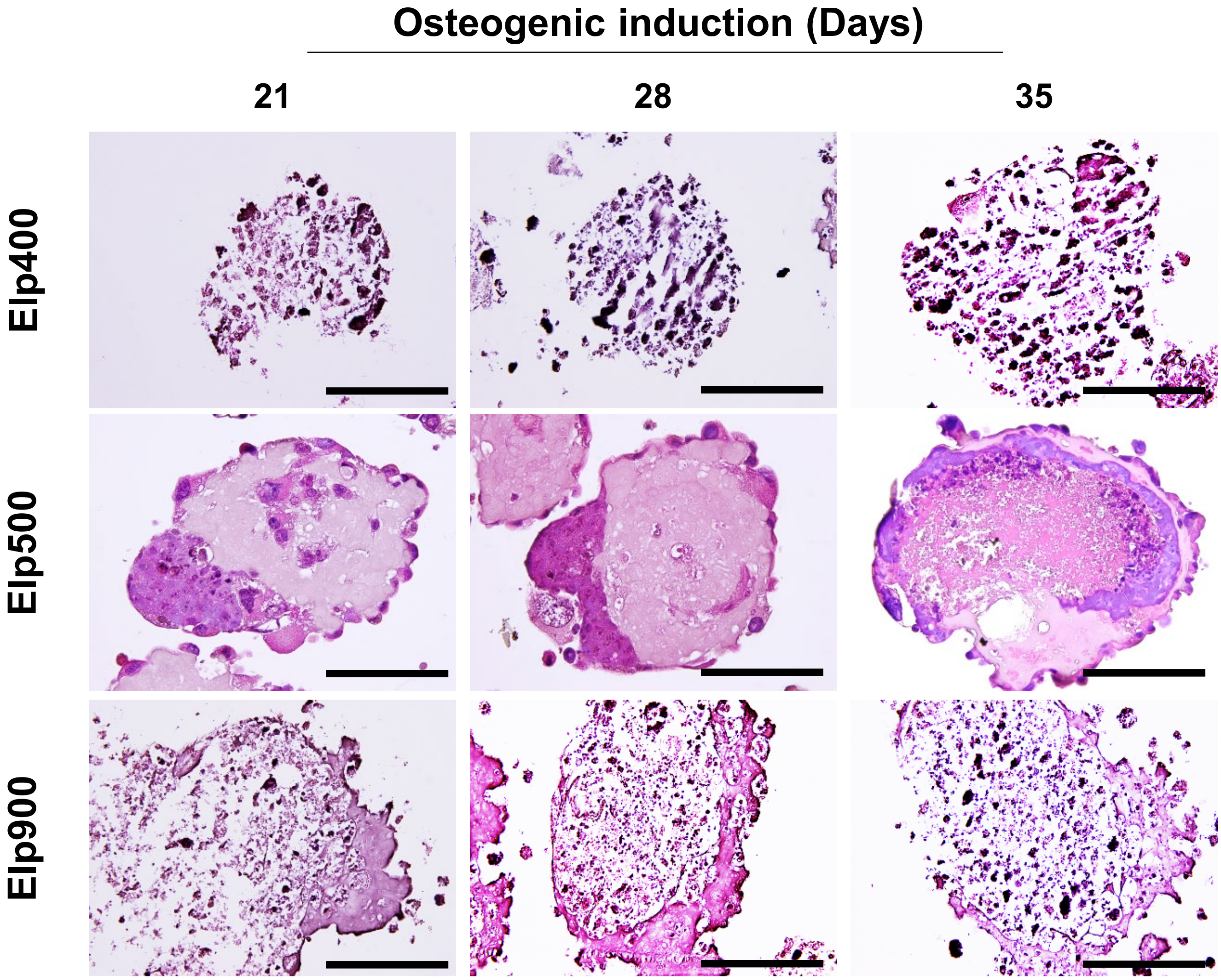

Supp Figure 2

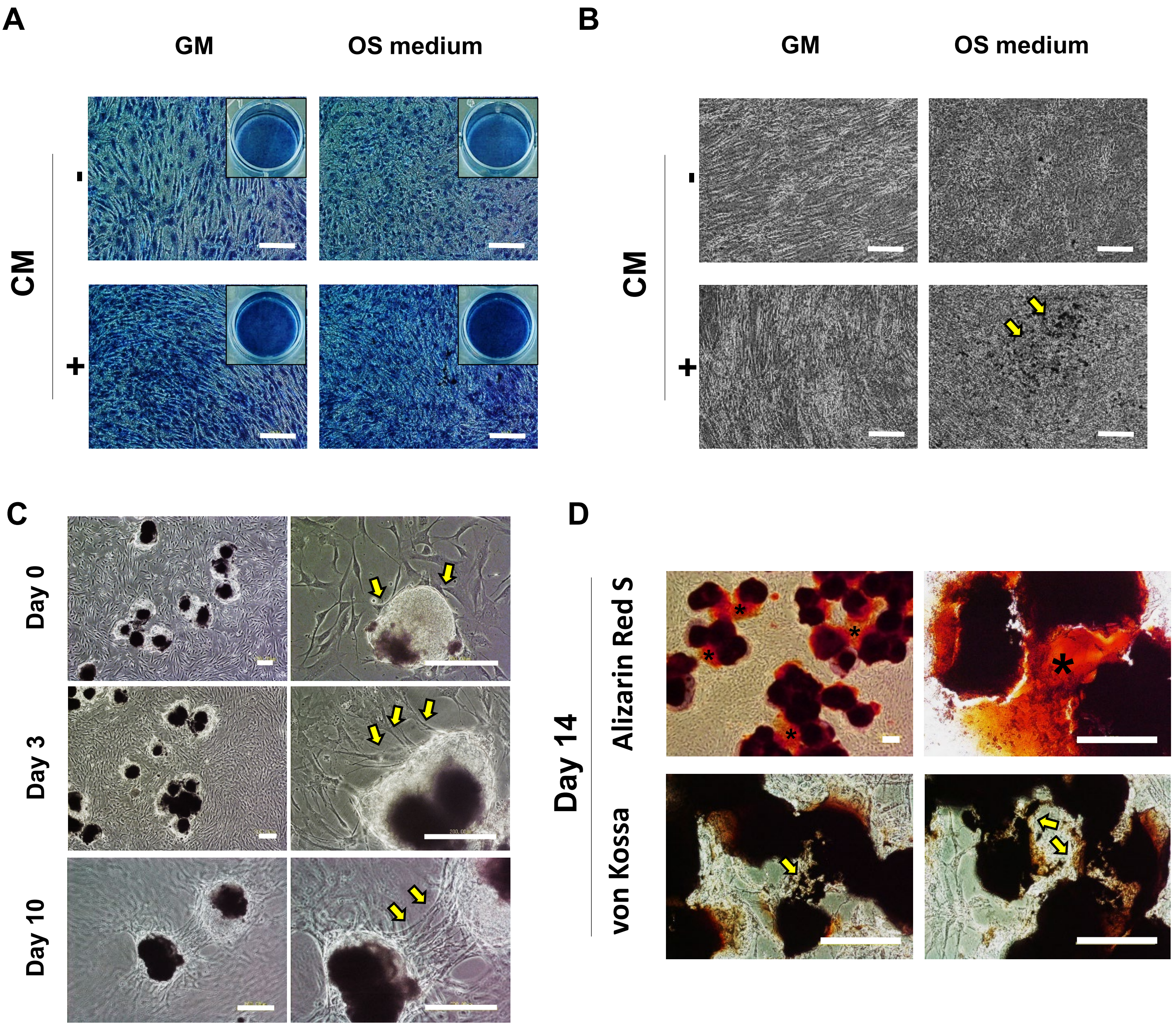

Supp Figure 3
